# P-914. Clinical Outcomes and Predictors of Mortality among Hospitalized HIV/ AIDS Adult Patients Diagnosed with Central Nervous System Infections in a National Tertiary Referral Hospital in the Philippines: A 5-year Retrospective Study

**DOI:** 10.1093/ofid/ofae631.1105

**Published:** 2025-01-29

**Authors:** Ma Jean Capulong Linsao, Dante P Bornales, Rontgene M Solante, Edna Edrada

**Affiliations:** San Lazaro Hospital, Calumpit, Bulacan, Philippines; San Lazaro Hospital, Manila, PH, Manila, National Capital Region, Philippines; San Lazaro Hospital, Calumpit, Bulacan, Philippines; San Lazaro Hospital, Calumpit, Bulacan, Philippines

## Abstract

**Background:**

HIV infection remains a significant global health concern despite the major advances in its understanding and treatment. Central nervous system (CNS) infections are common complications among HIV-infected patients. In the Philippines, limited local data on the clinical profile and outcome among HIV-infected patients with CNS infections is available.

Case Distribution of CNS Infections among HIV-infected patients in the Philippines

**Figure:**
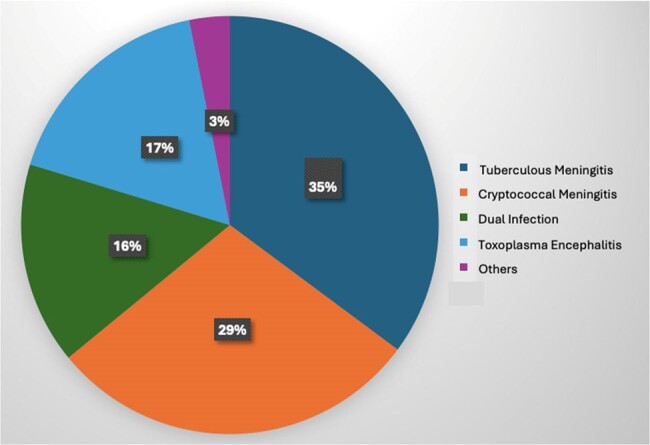

**Methods:**

This is a 5-year retrospective study of confirmed HIV-infected patients hospitalized and managed as CNS Infections at San Lazaro Hospital, Manila Philippines between 2018 and 2022. Demographics and clinical data were extracted from medical records and the association with clinical outcome and length of hospital stay were analyzed.

Incidence and Case Fatality Rate of CNS Infections among HIV-infected patients in the Philippines

**Figure:**
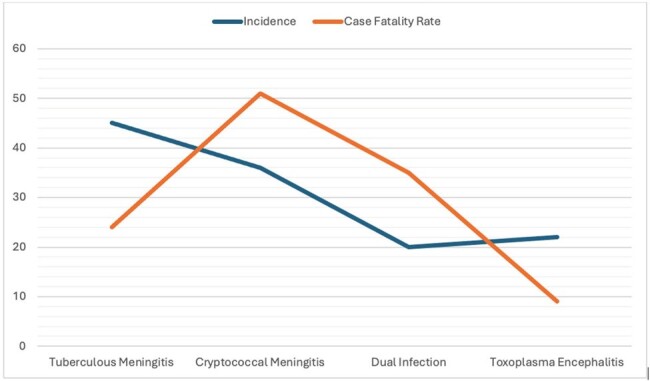

**Results:**

One hundred twenty-eight (128) HIV-infected patients, mostly male (95%), were included, with a mean age of 31 years, and a mean CD4 lymphocyte count of 56.8 (0-282) cells/mm^3^. Forty-one patients (32%) were newly diagnosed with HIV, and 51% (n=65) were not on antiretroviral therapy (ART). The mean duration of HIV diagnosis among those known to have HIV before admission was 1.4 years. The most common CNS infection was Tuberculous Meningitis (TBM) (35%), followed by Cryptococcal Meningitis (CM) (29%), and Toxoplasma Encephalitis (TE) (17%). The presence of dual CNS Infection was diagnosed among 20 patients (15.6%), of which a total of 16 (12.5%) had co-infection of TBM and TE, while 4 (3.1%) had co-infection of TBM and CM. The most common clinical manifestations were headache (87.5%), fever (61%), and sensorial changes (60%). The overall in-hospital mortality rate was 30.4% (n=39), with the highest case-fatality rate among those infected with Cryptococcal Meningitis at 53% (n=19). Among those diagnosed with CM, 23 patients (62%) had concomitant Cryptococcal Fungaemia. Those with CM and dual CNS infection had a longer hospital stay of >31 days ( >1 month).

**Conclusion:**

In the Philippines, the most common CNS infection among HIV-infected patients was Tuberculous Meningitis, followed by Cryptococcal Meningitis, and Toxoplasma Encephalitis. Early diagnosis of HIV infection with timely initiation of ART, together with an effective diagnostic and management approach to these conditions is needed to decrease the risk of further complications and death.

**Disclosures:**

**All Authors**: No reported disclosures

